# Analysis of the microvascular morphology and hemodynamics of breast cancer in mice using SPring-8 synchrotron radiation microangiography

**DOI:** 10.1107/S1600577517008372

**Published:** 2017-08-02

**Authors:** Masae Torii, Toshifumi Fukui, Masashi Inoue, Shotaro Kanao, Keiji Umetani, Mikiyasu Shirai, Tadakatsu Inagaki, Hirotsugu Tsuchimochi, James T. Pearson, Masakazu Toi

**Affiliations:** a Department of Breast Surgery, Graduate School of Medicine, Kyoto University, Kyoto, Japan; b Medical Imaging System Development Center, Canon, Tokyo, Japan; c Department of Diagnostic Imaging and Nuclear Medicine, Graduate School of Medicine, Kyoto University, Kyoto, Japan; d Research and Utilization Division, Japan Synchrotron Radiation Research Institute, Hyogo, Japan; e Department of Cardiac Physiology, National Cerebral and Cardiovascular Center Research Institute, Osaka, Japan

**Keywords:** tumor microvessels, hemodynamics, quantitative analysis, SR microangiography

## Abstract

Tumor vasculature is characterized by morphological and functional abnormalities. However, analysis of the dynamics in blood flow is still challenging because of limited spatial and temporal resolution. Synchrotron radiation (SR) microangiography above the *K*-edge of the iodine contrast agent can provide high-contrast imaging of microvessels in time orders of milliseconds. In this study, mice bearing the human breast cancer cell lines MDAMB231 and NOTCH4 overexpression in MDAMB231 (MDAMB231^NOTCH4+^) and normal mice were assessed using SR microangiography. NOTCH is transmembrane protein that has crucial roles for vasculogenesis, angiogenesis and tumorigenesis, and NOTCH4 is considered to be a cause of high-flow arteriovenous shunting. A subgroup of mice received intravenous eribulin treatment, which is known to improve intratumor core circulation (MDAMB231_eribulin). Microvessel branches from approximately 200 µm to less than 20 µm in diameter were observed within the same visual field. The mean transition time (MTT) was measured as a dynamic parameter and quantitative analysis was performed. MTT in MDAMB231 was longer than that in normal tissue, and MDAMB231^NOTCH4+^ showed shorter MTT [5.0 ± 1.4 s, 3.6 ± 1.0 s and 3.6 ± 1.1 s (mean ± standard deviation), respectively]. After treatment, average MTT was correlated to tumor volume (*r* = 0.999) in MDAMB231_eribulin, while in contrast there was no correlation in MDAMB231 (*r* = −0.026). These changes in MTT profile are considered to be driven by the modulation of intratumoral circulation dynamics. These results demonstrate that a SR microangiography approach enables quantitative analysis of morphological and dynamic characteristics of tumor vasculature *in vivo*. Further studies will reveal new findings concerning vessel function in tumors.

## Abstract 2

Synchrotron-radiation-based microvascular hemodynamic analysis was established in this study. Tumor vasculature analysis using this method showed unique characteristics of tumor blood flow *in vivo*.

## 1. Introduction   

Cancer requires the formation of tumor stroma for their growth and progression. The interactions between cancer cells and stromal cells like vascular cells in this microenvironment are essential for determining the properties of the tumor (Joyce & Pollard, 2009 ▸; Hanahan & Coussens, 2012 ▸). Tumor angiogenesis is one of the hallmarks of cancer in this sense. Some studies have been pivotal in characterizing the vascular morphology and hemodynamics of tumor formation as well as analyzing the molecular mechanisms of the tumor vasculature (Hanahan & Weinberg, 2000 ▸; Fukumura & Jain, 2007 ▸; Du *et al.*, 2008 ▸; Vakoc *et al.*, 2009 ▸; Carmeliet & Jain, 2011*a* ▸; Emblem *et al.*, 2013 ▸). Tumor vessels have highly irregular networks, immature vessel structures and abnormal blood flow as compared with normal vessels of normal tissues (Jain, 1988 ▸; Fukumura & Jain, 2008 ▸; Nagy *et al.*, 2009 ▸). It has become evident that microvessel density (MVD) is significantly increased in tumor tissues compared with adjacent normal tissues, but varies remarkably between tumors. Nevertheless, MVD often provides prognostic significance (Fox *et al.*, 1995 ▸; Uzzan *et al.*, 2004 ▸; Choi *et al.*, 2005 ▸). Antiangiogenic drugs such as anti-VEGF or anti-VEGF receptor antibodies are able to suppress tumor growth and progression in various types of cancers and improve progression-free survival time (Jain, 2001 ▸; Folkman, 2007 ▸). However, the therapeutic impact of such drugs on overall survival remains controversial (Bergers & Hanahan, 2008 ▸; Miles *et al.*, 2010 ▸; Robert *et al.*, 2011 ▸; Fakhrejahani & Toi, 2014 ▸; Jain, 2014 ▸). Therefore, from these limited findings, we need to know more about the characteristics and biology of tumor vasculatures (Jayson *et al.*, 2016 ▸).

Unorganized and heterogeneous intratumoral blood flow is known to be a key event that is driven by the abnormal tumor vasculature and further drives abnormal tumor vasculature development (Endrich *et al.*, 1979 ▸; Leunig *et al.*, 1992 ▸). Intratumoral blood flow is determined by multiple factors, not only by the immaturity of blood vessels but also by the physical stress imposed between cancer cell components and stromal cells in the microenvironment (Jain *et al.*, 2014 ▸). Obviously, the extracellular matrix plays a crucial role in this sense. Tumor vessels are also characterized as having immature vessel function. It is reported that tumor growth was actually decreased, whereas vessel densities were increased and vessels appeared to have poor perfusion leading to necrosis of tumor cells (Noguera-Troise *et al.*, 2006 ▸). Therefore, it is desirable to analyze the microvascular flow dynamics within the entire tumor; however, it has been very difficult to achieve this *in vivo*. Histological evaluation to achieve this research purpose has an important limitation that it is a static analysis.

In this study, we used synchrotron radiation (SR) microangiography, because SR above the *K*-edge of the iodine contrast agent can provide high-contrast imaging of microvessels in time orders of milliseconds (Sekka *et al.*, 2000 ▸; Tokiya *et al.*, 2004 ▸; Chien *et al.*, 2012 ▸). The brightness of SR allows greater X-ray penetration of thick tissues and greater image contrast. The very high degree of parallelism of SR does not produce a penumbra around the object and ensures blur-free images (Shirai *et al.*, 2013 ▸). In fact, it is possible to acquire microvessel images of vessels less than 20 µm in diameter (Yamashita, 2001 ▸) and to quantify the morphology and hemodynamics from the surface to deep parts of the tumor (Tokiya *et al.*, 2004 ▸; Liu *et al.*, 2010 ▸) in the same image. SR microangiography is unique in that conventional imaging modalities have, more or less, at least one limitation in their spatial resolution, observation depth (penetration) and or temporal resolution range (McDonald & Choyke, 2003 ▸). The resolution of ultrasound and contrast-enhanced magnetic resonance images (CE-MRI) are reported to be about around 100–200 µm. Micro-computed tomography (CT) and micro-MRI provide higher resolutions (<50 µm), three-dimensional images and quantitative evaluation of angiogenesis, but a serious limitation is the limited ability for real-time analysis of blood flow dynamics (Savai *et al.*, 2009 ▸; Kitahashi *et al.*, 2010 ▸; Missbach-Guentner *et al.*, 2011 ▸; Geffre *et al.*, 2015 ▸). SR microangiography is potentially useful to assess and quantify intratumoral blood vessel morphology and blood flow dynamics *in vivo* more precisely, and with high resolution, spanning several centimeters visualization depth.

Using SR microangiography, we visualized the tumor microvasculature in mouse models bearing human tumor xenografts, to assess the morphological characteristics of microvessels and the hemodynamics of tumor blood flow quantitatively. To determine the effect of chemotherapy treatment with eribulin mesylate, which is known to improve intratumoral blood perfusion by vascular remodeling (Funahashi *et al.*, 2014 ▸), we treated tumors with eribulin every seven days. Furthermore, to determine whether the vascular shunts in tumors have any effect on arterial microvascular flow, we manipulated NOTCH4 overexpression tumors. NOTCH is transmembrane protein that has crucial roles for vasculo­genesis, angiogenesis and tumorigenesis, and NOTCH4 is considered to be a cause for high-flow arteriovenous shunting in arteriovenous malformation (Murphy *et al.*, 2012 ▸, 2014 ▸). Normal tissue, xenograft tumors (MDAMB231), tumors treated with eribulin (MDAMB231_eribulin) and NOTCH4 overexpression tumors (MDAMB231^NOTCH4+^) were evaluated quantitatively.

Our results reveal the characteristic properties of the tumor microvessels in mouse models with human tumor xenografts.

## 2. Materials and methods   

### 2.1. Ethical approval   

All animal experiments were approved by the Animal Research Committee of Kyoto University and the local Animal Ethics Committee of SPring-8, and performed according to the guidelines governing animal care in Japan.

### 2.2. Cell lines   

The human breast cancer cell line MDAMB231 was obtained from the stock of the Department of Breast Surgery, Kyoto University, from the American Type Culture Collection (Rockville, MD, USA). Cells were maintained in RPMI1640 medium supplemented with 10% FBS and 100 units ml^−1^ of penicillin G and 100 µg ml^−1^ of streptomycin. All cell lines were maintained in a 37°C humidified incubator with 5% CO_2_.

### 2.3. Construction of NOTCH 4 expression vector   

NOTCH4 was generated by polymerase chain reaction (PCR) using the 5′ primers including artificial Kozak sequence (gcc gcc acc atg cag ccc cct tc ctg c) and the common 3′ primer: gga gga gag ggt aaa aaa tag to cDNA clone NOTCH4 ORF (accession: BC140782; clone ID: 9021650, MHS1768-213245964, Thermo). In preparation for cloning, the PCR product was phospho­rylated by ProK, and ligated into the pMscvPuro proviral vector.

The NOTCH4 coding sequence was excised by XhoI (Takara Bio, Otsu, Japan) and EcoRI (Takara Bio, Otsu, Japan) and inserted into pMscvPuro (Clontech, CA, USA). All plasmids were sequenced over their cloning junctions to verify integrity.

### 2.4. Gene transfection   

Cells were transfected with a pMscvPuro vector in RPMAI1640/10% FBS containing 0.2 µg ml^−1^ puromycin. Breast cancer cells were also infected with the retrovirus containing GFP, which was obtained by transfecting pRS-puro-GFP plasmid in G3T-hi cells using a Retrovirus Packaging kit Ampho (Takara Bio, Shiga, Japan), and GFP+ cells were confirmed with fluorescence activated cell sorting ARIA (BD Biosciences, Franklin Lakes, NJ, USA). NOTCH4 expression was confirmed with western blotting following a standard protocol using primary antibodies against human NOTCH4 (abcam, ab-33163 1:300) conjugated to horseradish peroxidase secondary antibody. Blots were developed using enhanced chemiluminescence (Supersignal West Pico, Pierce).

### 2.5. Xenograft model   

Cancer cell suspensions and BD Matrigel (BD Biosciences, BD Matrigel TM Basement Membrane Matrix) were transplanted into the mammary fat pads of female mice (NOD.CB17-Prkdcscid/, Charles River Laboratories Japan, Inc., 1 × 10^6^ cells per animal) (Proia & Kuperwasser, 2006 ▸).

### 2.6. Reagents and treatment protocol   

Xenograft mice were randomized into two groups: a treatment group and a control group. In the treatment group, mice were treated with 1.5 µg g^−1^ eribulin mesylate (Eisai, Tokyo, Japan) dissolved in normal saline. Treatments started when tumor length reached approximately 7 mm (14–21 days after cell transplantation), with eribulin administration *via* a tail vein every 7 days. Tumor length (mm) and mouse weight (g) were measured once a week. Tumor volume was calculated as [width (mm)^2^ × length (mm)]/2 (Funahashi *et al.*, 2014 ▸). After three weeks of treatment, mice in the treatment group were classified according to tumor volume.

### 2.7. Anesthesia and surgical preparation   

Mice were systemically anesthetized by sodium pentobarbital (60 mg kg^−1^ i.p.) and intradermal injections of 1% lidocaine hydro­chloride monohydrate were delivered at the incision site at the neck. Following a tracheotomy the trachea was intubated and lungs were ventilated with a mouse ventilator (10 µl g^−1^ tidal volume and 130–150 breaths min^−1^; Hugo-Sachs Minivent type 845, Harvard Instruments) (Sonobe *et al.*, 2011 ▸).

**Figure 1:**
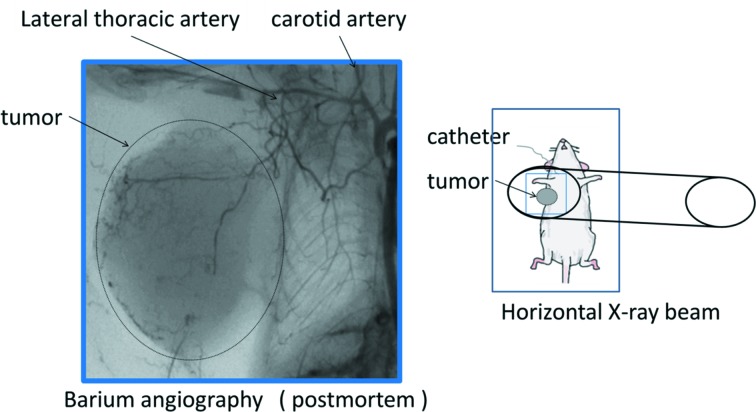
(Right) Schematic drawing showing the anesthetized mouse secured to an acrylic sheet mounted in the path of the horizontal X-ray beam for synchrotron microangiography experiments. (Left) A *post mortem* barium angiogram showing the vessel architecture around and within a tumor transplanted into the mammary fat pad in a mouse. A branch of the lateral thoracic artery from the carotid artery supplies blood to the tumor.

In order to better understand the entire target tumor blood vessels, in pilot studies we visualized the tumor blood vessels *in situ* in mice by *post mortem* barium angiography using a microfocus laboratory X-ray angiography system (MFX-80H, Hitex Co. Ltd, Osaka, Japan) (Fig. 1 ▸). We confirmed that a branch of the lateral thoracic artery supplies blood to the tumor as shown in Fig. 1 ▸. A catheter was introduced into a carotid artery or a brachial artery for iodinated contrast media administration. Skin above the tumor was cut and made into a flap for exposing the tumor. Tumor was delineated with a wire of 50 µm diameter and covered with Vaseline to prevent drying. The mouse was securely restrained on an acrylic sheet which was placed vertically within the beam so that the X-ray passed through the mouse and the acrylic sheet (sagittal plane). Throughout the experimental protocol, body temperature was maintained with a thermostatically controlled heating pad.

### 2.8. Experimental protocol   

Experiments were performed as in previous reports (Schwenke *et al.*, 2007 ▸; Sonobe *et al.*, 2011 ▸), with modifications for our purpose. Each mouse was set in the vertical position and the tumor was positioned in alignment with the center of the 5 mm × 5 mm imaging field. An iodinated contrast agent (0.15 ml bolus, Iomeron400 delivered at 11 ml min^−1^; Eisai) was injected *via* the carotid artery cannula with a microinjection pump (PHD Ultra 4400; Harvard Apparatus). Image acquisition was initiated 1 s before iodine injection, and 500 frames (50 frames s^−1^ × 10 s) recorded for each scan. Artificial ventilation was briefly stopped during imaging (Pearson *et al.*, 2013 ▸). Mice were given at least 5 min to recover from each bolus injection of contrast media.

### 2.9. SR microangiography   

The tumor microcirculation was visualized using SR microangiography at the BL20B2 beamline of SPring-8 facility, Hyogo, Japan. The validation of the accuracy of SR for visualizing circulation *in vivo* has previously been described (Tokiya *et al.*, 2004 ▸; Shirai *et al.*, 2013 ▸). SR microangiography was performed with monochromatic X-rays just above the iodine absorption *K*-edge energy (33.17 keV) at 33.2 keV for maximal contrast. At this energy the highest absorption contrast images of the iodine contrast media in the lumen of vessels are produced while bone and soft tissue contrast is low. X-rays were detected by a dedicated Orca camera (ORCA_Flash4.0 CMOS camera; Hamamatsu Photonics KK, Shizuoka, Japan) with a BM-2 optics (fluorescent screen and lens system; Hamamatsu Photonics KK, Shizuoka, Japan). X-ray flux at the mouse position was around 1 × 10^9^ photons mm^−2^ s^−1^ at an energy of 33.2 keV. Therefore, a typical dose of radiation was 3.3 mGy per image with an exposure time of 50 ms.

### 2.10. Image analysis   

The computer-imaging analysis program *ImageJ* (1.47t) (National Institutes of Health) was used to enhance contrast and the clarity of angiogram images. Background images were generated by averaging images from slices 1 to 40, before contrast media injection. To enhance vessel images, a Gaussian blurred background image was subtracted from each image. *ImageJ* was also used to evaluate the vessel diameter and depict the intensity of contrast media at the region of interest (ROI) on the blood vessel.

### 2.11. Quantification of morphology and hemodynamics in microvessels   

**Figure 2:**
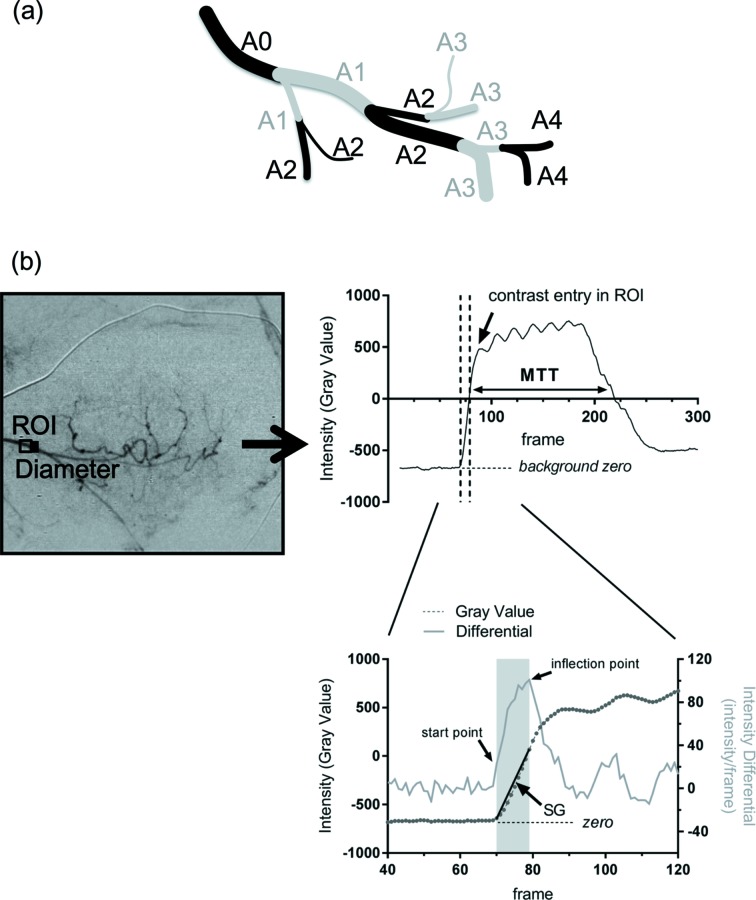
(*a*) Categorization of arterial branching order. After the main vessel (A0) branch, the number of vessels increases with each successive branch. (*b*) Setting of the region of interest (ROI) in a temporally subtracted microgram at A0 for diameter measurement, and depiction of the moving averaged intensity profile over the first 300 frames (upper right panel) of the subtracted image, and the time intensity curve of the contrast media in the ROI (line with circles) alongside the intensity differential plot (pale gray line) of the time intensity curve (lower panel). The shaded region indicates the start and finish of the frames used for determination of the gradient of the slope (SG), which was used as the index of arterial blood flow at each branch point. The intensity average of frames 1–40 is indicated as the background zero intensity under the time intensity curve (horizontal black dashed lines). Mean transition time (MTT) was calculated as the duration that the intensity was sustained at half the maximum intensity gray level as a second estimate of blood flow.

Our microangiogram analysis of blood flow was restricted to the arterial phase. The vessel diameter, the number of branches in the microvasculature, and mean arterial transition time (MTT) were the key parameters obtained from the SR microangiograms. Vessel branches were categorized according to branching level, with the number increasing with each successive branch from the main vessel defined as A0 (Fig. 2*a* ▸) (Savai *et al.*, 2009 ▸). The number of branches was counted. Each ROI was set at the segment midpoint of branches of the blood vessel and a time intensity curve (TIC) of the contrast media was then smoothed by using a moving average of ten frames (Fig. 2*b* ▸). Vessel diameter was manually measured in the middle of segments perpendicular to the vessel on the downstream side of the ROI. The gradient of the slope of the TIC was calculated by the difference of intensity divided by the difference in frame number between the starting point and flexion point as an index of arterial flow (Fig. 2*b* ▸). We defined the start point as the time point as the second consecutive frame in which the ROI intensity first exceeded twice the average intensity of frames 1 to 40 and the end point as the point at which the intensity differential first declined. We calculated the MTT at each ROI set on each of the vessels as the duration for which the gray level intensity was more than half its maximum level of intensity relative to the gray level before contrast entry (Fig. 2*b* ▸). We compared the MTT between the three groups (normal tissue, MADMB231 and MDAMB231^NOTCH4+^) for each vessel segment.

### 2.12. Data analysis   

One angiogram series per mouse was selected for analysis and angiogram series with major motion or image artifacts were excluded. All statistical analyses were conducted with JMP (SAS Institute Inc. version 10). Results are presented as mean ± standard deviation (SD) for MTT and as median (minimum–maximum) for vessel diameter. A *p* value of 0.05 was predetermined as the level of significance for all statistical analysis.

## 3. Results   

### 3.1. Image acquisition by SR microangiography   

Representative angiographic images of the tumor vessels in the xenograft model and mammary fat pads obtained by SR microangiography are shown in the supporting movies (supporting movies can be found with this article online at https://doi.org/10.1107/S1600577517008372). These movies show the blood perfusion in normal mammary fat pads and that in a tumor. In normal tissue, blood flow perfusion spread smoothly from proximal vessel to the capillaries. Blood flow was gradually transferred from arteries to veins and the background tissue was homogeneously stained following transit from the arteries. The vessel structure was smooth. On the other hand, perfusion in the representative tumor example showed irregular vessel structure including tortuous form, a high degree of branching and irregular vessel width. Contrast media in proximal vessels were retained after staining the capillary beds in the tumor example. Perfusion towards the capillaries was heterogeneous showing patchy dark pooling areas. These movies displayed the characteristic differences of the microvasculature between normal tissue and the tumors.

### 3.2. Morphological analysis   

**Figure 3:**
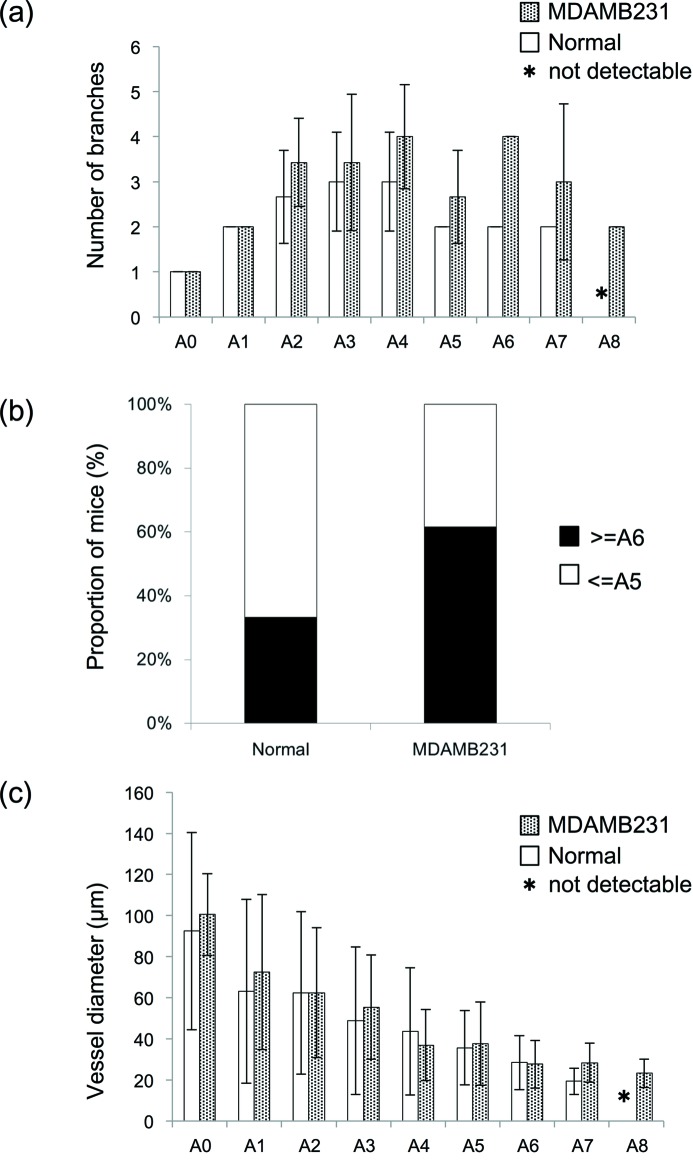
Comparison of morphological parameters of microvessels between normal and MDAMB231 mice. (*a*) The number of branches of each branch order is relative to the A0 origin and shows branches as an A0 ratio. There were A8 branches in MDAMB231, but only A7 branches in normal tissue (normal: *n* = 6; MDAMB231: *n* = 7). (*b*) The proportion of mice with more than A6 branches and less than A5 branches. Mice with more than A6 branches were 2 out of 6 (33.3%) in normal mice and 8 out of 13 (61.5%) in MDAMB231. (*c*) There was no significant difference in vessel diameter (normal: *n* = 6; MDAMB231: *n* = 13). The asterisk (*) indicates that a segment was not observed. The error bars show SD of the mean branch ratio in (*a*) and mean absolute internal diameter in (*c*). The absence of error bars in (*a*) indicates that all animals showed the same change in branch ratio and therefore there is zero error.

Our microangiogram images enabled visualization of microvessel branches from approximately 200 µm to less than 20 µm in diameter within the same visual field. Contrast media were visualized throughout the networks of tumor-feeding arteries, capillaries and veins. The number of branches from the main vessel A0 to the capillary bed was nine (A0–A8) in MDAMB231 and eight (A0–A7) in normal tissue (Fig. 3*a* ▸); however, the vessels in MDAMB231 were branched more than that in normal tissue. The normal branching pattern which branched most at A3 and A4 was reduced after A5, whereas the number of branches in the MDAMB231 was increased from A3 to A7 and was sometimes reduced at A5. The number of mice with more than A6 branches was 2 out of 6 (33.3%) in normal mice and 8 out of 13 (61.5%) in MDAMB231-bearing mice (Fig. 3*b* ▸). The diameter of the vessels in MDAMB231 and normal tissue was similar among the series, at 37.1 µm (9.2–140.1 µm) and 43.1 µm (13.0–173.0 µm) [median (minimum–maximum)] (Fig. 3*c* ▸). The minimum diameter was measured immediately before the capillary beds, as contrast media appear diffuse in the capillary beds and were therefore unmeasurable. There were no significant differences between MDAMB231 and normal tissue for vessel diameter categorized by branching order.

### 3.3. Hemodynamic analysis   

**Figure 4:**
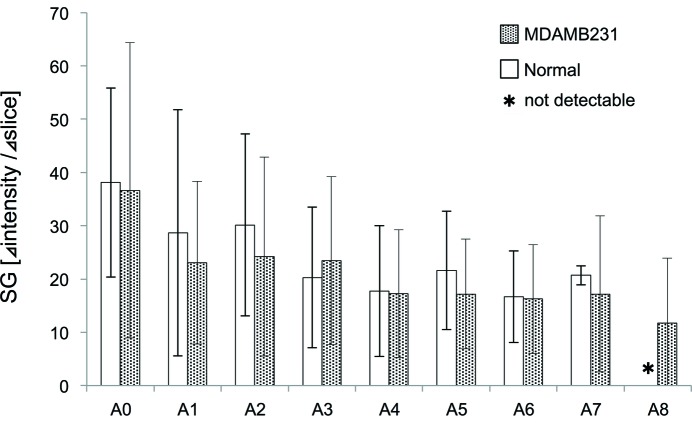
Comparison of slope gradient (SG) between normal and MDAMB231 tissue. There was no significant difference in SG. The asterisk (*) indicates that a segment was not observed. The error bars show SD of the mean.

**Figure 5:**
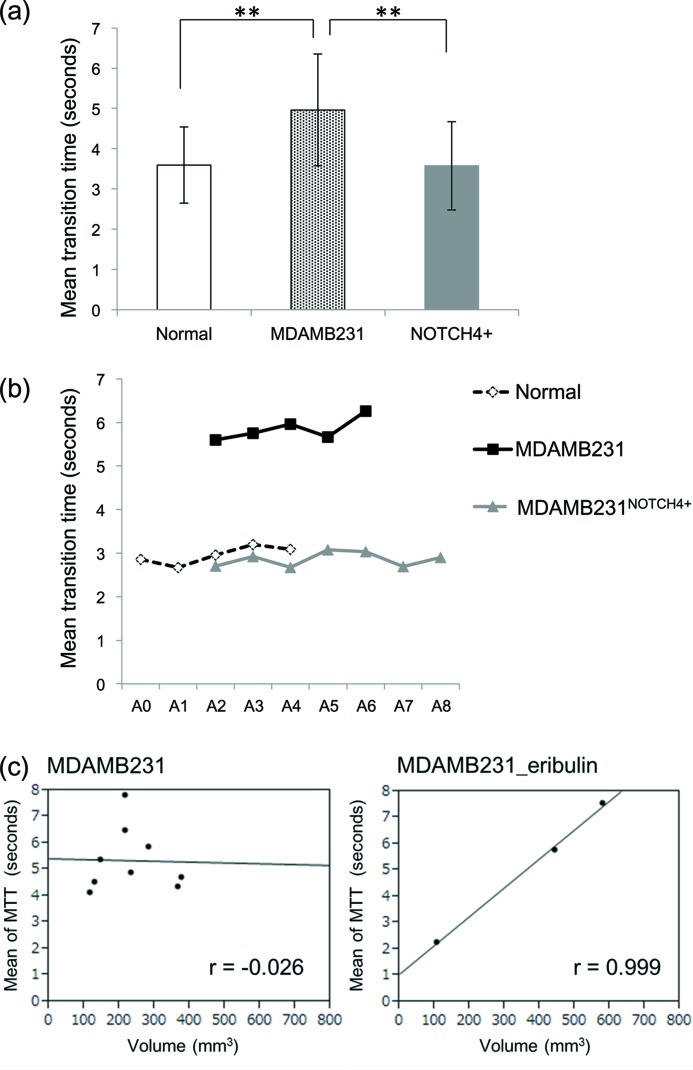
(*a*) Group mean of the MTT of the vessels in normal tissue (*n* = 4), MDAMB231 (*n* = 9) and MDAMB231^NOTCH4+^ (*n* = 8). The MTT was longer in MDAMB231 than in normal tissue. In mice bearing MDAMB231^NOTCH4+^, the MTT significantly decreased (MDAMB231: 5.0 ± 1.4 s; normal: 3.6 ± 1.0 s; MDAMB231^NOTCH4+^: 3.6 ± 1.1 s). (*b*) Representative plots of MTT in each group. MTT shows a similar duration in each branch in all groups. (*c*) Left: no correlation was observed between the MTT of the vessels and tumor volume in MDAMB231 (*n* = 9). Right: correlation between the MTT of the vessels and tumor volume following three weeks of eribulin treatment (MDAMB231_eribulin) (*n* = 3). With the enlargement of volume, MTT significantly increased. ** *p* < 0.05. The error bars show SD of the mean. *r*: Pearson’s correlation coefficient.

The blood flow in tumors appeared to perfuse slower and more irregularly than that in normal tissue. Further, there seemed to be contrast media remaining in tumor microvessels at the later phase of the angiogram series of 10 s. However, in our study, Fig. 4 ▸ shows that the gradient of the slope at each branching point was not significantly different between MDAMB231 and normal tissue. On the other hand, as shown in Fig. 5(*a*) ▸, the mean MTT was significantly longer in MDAMB231 (5.0 ± 1.4 s) than that in normal tissue (3.6 ± 1.0 s), indicating that perfusion through the microvessels was slower in MDAMB231 (*p* < 0.05, nonparametric Wilcoxon test). In contrast, the mean MTT of MDAMB231^NOTCH4+^ (3.6 ± 1.1 s) was shorter than that of MDAMB231. Therefore, vessel structure appears to affect the flow dynamics in tumors. Fig. 5(*b*) ▸ shows the representative plots of MTT in each group. MTT showed similar durations in each branch in all groups. Furthermore, there was no correlation between tumor volume and mean MTT in MDAMB231, but MDAMB231_eribulin, treated with eribulin over three weeks, showed a direct correlation between tumor volume and MTT (Fig. 5*c* ▸). MDAMB231_eribulin with an enlarged volume of about 500 mm^3^ showed longer MTT. However, this result was from a small number of mice and preliminarily in nature. Therefore, further examination is needed.

### 3.4. NOTCH4 analysis   

**Figure 6:**
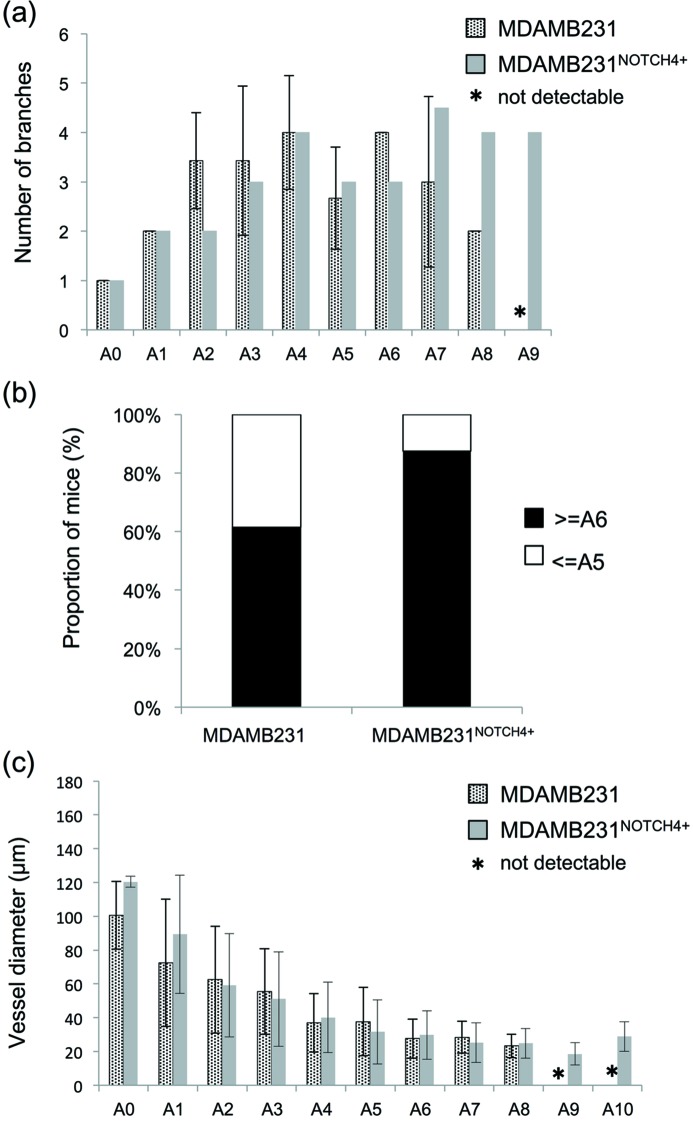
Comparison of morphological parameters of microvessels between tumors prior to treatment (MDAMB231) and NOTCH4 overexpression tumor (MDAMB231^NOTCH4+^) tissue. (*a*) The number of branches of each branch order is shown relative to the A0 origin (MDAMB231: *n* = 7; MDAMB231^NOTCH4+^: *n* = 2). (*b*) The proportion of mice with more than A6 branches and less than A5 branches is presented. Mice with more than A6 branches were 8 out of 13 (61.5%) in MDAMB231-bearing mice and 7 out of 9 (87.5%) in MDAMB231^NOTCH4+^-bearing mice. (*c*) The vessel diameter of MDAMB231 (*n* = 13) was 37.1 (9.2–140.1) and 27.9 (7.8–167.2) µm in MDAMB231^NOTCH4+^ (*n* = 8) (median: minimum–maximum). There were A8 branches present in all tumors, but more branching orders (A9, A10) were present in MDAMB231^NOTCH4+^ tissue. There were no significant differences in vessel diameter. The asterisk (*) indicates that a segment was not observed. The error bars show SD of the mean, except in (*a*) where MDAMB231^NOTCH4+^ counts were based on two mice only and therefore error bars are omitted.

The number of the branches in MDAMB231^NOTCH4+^ was more than MDAMB231 at A7, A8 and A9 (Fig. 6*a* ▸). The branches increased in MDAMB231^NOTCH4+^ (Fig. 6*a* ▸). The number of mice with more than A6 branches was 8 out of 13 (61.5%) in MDAMB231-bearing mice and 7 out of 9 (87.5%) in MDAMB231^NOTCH4+^-bearing mice (Fig. 6*b* ▸). The diameter of the vessels in MDAMB231 and MDAMB231^NOTCH4+^ was 37.1 µm (9.2–140.1 µm) and 27.9 µm (7.8–167.2 µm) [median (minimum–maximum)] (Fig. 6*c* ▸). There were no significant differences in vessel diameter.

## 4. Discussion   

In this study we analyzed the characteristics of tumor vascular structure and their hemodynamics quantitatively. As expected, our high-resolution angiograms of the tumor vasculature revealed abnormal structures such as tortuous irregular shape, a high degree of branching and irregular vessel width. In addition, the intratumoral perfusion up to the capillary bed was heterogeneous, showing patchy dark and pooling areas. Moreover, contrast medium was retained in the proximal vessels in tumors after passage through the capillary beds, which was remarkably different from the blood perfusion in normal mammary fat pads.

In fact, SR microangiography provided high-resolution images where we could identify microvessels from approximately 200 µm to less than 20 µm in diameter within the same visual field. The tumor-associated microvessels showed complicated branching patterns and more branches, as compared with normal microvessels. The vessel diameter in normal tissues became smaller proportionally as branching progressed, but those of tumor vessels did not. In tumors, microvessels did not arborize evenly, and the diameter, in some cases, was bigger in the distal side rather than the proximal side (Fig. 2*c* ▸). The branching proximal to capillary beds was sometimes more frequent in tumor microvessels than in normal microvessels (Figs. 2*a*, 2*b* ▸). These morphological characteristics appear to represent the abnormal characteristics of tumor microvessels (Leunig *et al.*, 1992 ▸; Carmeliet & Jain, 2000 ▸).

The most important novel finding in this study was that MTT, as an index of the hemodynamics state, was longer in MDAMB231 than in normal tissue. Thus blood flow took longer to enter the capillary bed through the tumor microvessels. Since MTT is effectively the time taken for contrast entry and clearance of the arterial vessel on the TIC, a longer MTT may reflect a more extensive vascular branching network of high vascular resistance distal to the main arterial branches, and therefore slow flow, as well as high interstitial pressure (Ridolfi *et al.*, 2012 ▸). In previous studies, tumor vasculature has been shown to have highly irregular dynamics, *i.e.* flow stasis (Jain, 1988 ▸; Leunig *et al.*, 1992 ▸; Fukumura *et al.*, 2010 ▸). Tumor vessels are sometimes compressed by stromal pressure and blood flow is unstable spatially and temporally (Jain *et al.*, 2014 ▸). Alternatively, as we only observed a trend for more branches in the A5–A7 region of the tumor network it might also be possible that the greatest resistance might lie within the smallest precapillary arterioles, which are below the *in vivo* imaging resolution with this SR imaging approach. However, our results are more likely to be showing the complex nature of flow patterns in tumor tissues. MTT was similar in each branch segment in the arterial phase (Fig. 5*b* ▸). Furthermore, the correlation between MTT and tumor volume was not seen in untreated tumors, but MTT became more prolonged in association with tumor progression in some of the eribulin-treated mice. A clear correlation between MTT and tumor volume indicates that MTT became shorter in mice in which eribulin was able to suppress tumor growth relative to mice in which tumor growth was not suppressed by treatment (Fig. 5*c* ▸). This might explain the ‘normalization’ effect of some chemotherapy agents on vessel remodeling, including improved perfusion, oxygenation and the efficacy of chemotherapy (Carmeliet & Jain, 2011*b* ▸).

We also tried to visualize the vascular shunts present in tumors as an exploratory study with a NOTCH4 overexpression tumor xenograft model. NOTCH4 is reported as one of the key molecules for arteriovenous malformation, and has been shown to be associated with shunts or enlargement of vessels formation in brain tissue (Murphy *et al.*, 2012 ▸, 2014 ▸). In our study, the MTT of MDAMB231^NOTCH4+^ was shorter than non-transfected tumors (MDAMB231) (Fig. 5*a* ▸). The number of the branches was increased in MDAMB231^NOTCH4+^ (Fig. 6 ▸). This suggests that increased vascular beds required more blood flow and flow perfused rapidly. The increase in blood flow suggests that more of the formed vessels in the tumor were open and well perfused due to shunt-like behavior. However, it must be stated that our results are limited by the small number of mice and observation for the arterial phase only; therefore, further examination is needed. In the future, analysis of contrast motion in the microvessels of tumors with approaches such as particle velocimetry might shed further light on the blood flow patterns in these precapillary vessels.

SR microangiography enabled the quantification of *in vivo* microcirculation at high resolution (approximately 10 µm) not only around the tumor surface but also inside the tumor volume up to 1 cm in depth. The spatial resolution of *in vivo* micro-CT with contrast media utilizing non-synchrotron sources has been shown to be about 35 µm. Micro-MRI has a high spatial resolution of 10 µm but poor temporal resolution (McDonald & Choyke, 2003 ▸). While confocal microscopy and multiphoton microscopy enable microstructures to be visualized within approximately 100 nm, and scanning electron microscopes have a resolution of a few nanometers, these images are limited to superficial areas or narrow planes of focus within a limited depth, and scanning electron microscopy is not suitable for *in vivo* measurements (McDonald & Choyke, 2003 ▸; Vakoc *et al.*, 2009 ▸; Fukumura *et al.*, 2010 ▸). SR microangiography achieves both high resolution and imaging of deep tissues.

Angiography is an effective technique for depicting lumen and microstructural details of vessels, but it has some limitations as well. We acquired images that were two-dimensional and not three-dimensional. Therefore, these two-dimensional images cannot provide information on the depth of vessels that are non-uniform in shape, which makes it difficult to interpret the functions and complete structures of vessels even with overlapping images of the region of interest. Hoshino *et al.* (2011 ▸) split the monochromatic beam upstream of the sample with a silicone crystal to produce stereogram images, which enabled the authors to recognize the three-dimensional structure of *in situ* blood vessels. We also attempted the acquisition of two stereographic angiograms separated by a small angle difference in the beam path through the tumors. These preliminary results highlight the possibility of recognizing vessel structure in still images and movies, without much difficulty. However, the overlap with tissue features affected the quality of two-dimensional images of the vasculature and thus we did not successfully obtain TIC curves with the stereography approach. Further refinements may remedy this problem for three-dimensional-like acquisition.

Quantitative analysis of tumor vasculature has focused on biomarkers in several types of cancers including brain tumors (Jayson *et al.*, 2016 ▸). However, it is equally important to develop novel methodologies to assess the characteristics of the tumor vasculature. We characterized the tumor circulation in several ways, but further analysis approaches need to be developed. The heterogeneity of tumor microvessels may be one of the important issues that needs further consideration. In our study, the slope gradient (SG), which is the amount of intensity change per time, did not show significant difference between the groups in the arterial phase, but it may be useful to measure the rate of intensity change in the venous phase and the combined analyses with MTT and SG would give us further information on the tumor vessel properties (Leunig *et al.*, 1992 ▸; Savai *et al.*, 2009 ▸).

Although this application of SR imaging methodology might be preliminarily, it shows promise for revealing the morphological and functional characteristics of tumor microvessels *in vivo* and it may be applicable for other tumor models. The information obtained from studies that utilize SR microangiography has the potential to help us to understand the differences between normal and abnormal vasculature *in vivo*. In particular, it provides an important means of assessing blood flow dynamics in tumors. With appropriate consideration of radiation exposure, it is now possible to observe alterations in vascular morphology and intratumoral micro-hemodynamics during tumor progression sequentially over time (Cai, He *et al.*, 2012 ▸; Cai, Sun *et al.*, 2012 ▸). Further studies are needed to develop new markers of abnormal tumor microvessels. In the near future, we will focus on characterizing the regression of tumor vasculature driven by anticancer treatments with these techniques.

## 5. Conclusion   

We have shown that the analysis of microvascular hemo­dynamics with a SR microangiography approach is useful for analyzing the characteristics of tumor microvasculature. These preclinical findings may help to understand vessel function in tumors.

## Supplementary Material

Typical microangiogram movies showing an image field of 5 mm x 5 mm square. The study area was delineated by a wire of 50 micrometer diameter. These videos were acquired for 10 s at 50 frames per second. Movie 1: perfusion in normal tissue.. DOI: 10.1107/S1600577517008372/ay5497sup1.avi

Typical microangiogram movies showing an image field of 5 mm x 5 mm square. The study area was delineated by a wire of 50 micrometer diameter. These videos were acquired for 10 s at 50 frames per second. Movie 2: perfusion in a tumor.. DOI: 10.1107/S1600577517008372/ay5497sup2.avi
